# Successful Exsection of Inferior Dental Nerve for Obstinate Neuralgia; Bone Grafting

**Published:** 1883-10

**Authors:** W. Gardner


### Successful Exsection of Inferior Dental Nerve for Obstinate Neuralgia; Bone Grafting.

Mr. M., aged 60, consulted me on November 9, 1882, for persistent pain on the alveolar border of the lower jaw (right side) midway between the angle and the symphysis. The pain had been present for five years, with exacerbations which rendered his life unbearable. There were no teeth in the lower jaw except the incisors. I proposed to him to cut down and remove a piece of the inferior dental nerve, and to this he willingly assented. On the next day, however, I tried the effect of dividing the inferior dental nerve at its entrance into the inferior dental foramen. As this failed to give any relief, I made an incision about two inches long, parallel to the lower border of the jaw, dividing the facial artery, which I tied. I then divided the periosteum, and separated it sufficiently from the bone to allow of the application of the trephine. A trephine, half an inch in diameter, was then applied over the site of the nerve, and after cutting down about half an inch, the bone was elevated, and a slight additional application of the instrument opened the inferior dental canal, where the nerve was seen lying. I then removed half an inch of the nerve, and at the suggestion of Drs. Sterling and Jay, I replaced the plug of bone to try an experiment in bone-grafting, and drew the divided edges of the periosteum over it. The operation was followed by complete relief to all the symptoms, and the wound was perfectly healed in a week, with the exception of a small opening where one of the ligatures came out. The plug which was replaced has not caused the slightest irritation, and may now reasonably be supposed to have become reunited to the rest of the bone.—*W. Gardner, M.D., in Australian Med. Journal.*

